# A Multi-Omics View of Maize’s (*Zea mays* L.) Response to Low Temperatures During the Seedling Stage

**DOI:** 10.3390/ijms252212273

**Published:** 2024-11-15

**Authors:** Tao Yu, Jianguo Zhang, Xuena Ma, Shiliang Cao, Wenyue Li, Gengbin Yang

**Affiliations:** 1 Maize Research Institute, Heilongjiang Academy of Agricultural Sciences, Harbin 150086, China; 2 Key Laboratory of Biology and Genetics Improvement of Maize in Northern Northeast Region, Ministry of Agriculture and Rural Affairs, Harbin 150086, China; 3 Key Laboratory of Germplasm Resources Creation and Utilization of Maize, Harbin 150086, China

**Keywords:** low-temperature, maize, metabolome, transcriptome

## Abstract

Maize (*Zea mays* L.) is highly sensitive to temperature during its growth and development stage. A 1 °C drop in temperature can delay maturity by 10 days, resulting in a yield reduction of over 10%. Low-temperature tolerance in maize is a complex quantitative trait, and different germplasms exhibit significant differences in their responses to low-temperature stress. To explore the differences in gene expression and metabolites between B144 (tolerant) and Q319 (susceptible) during germination under low-temperature stress and to identify key genes and metabolites that respond to this stress, high-throughput transcriptome sequencing was performed on the leaves of B144 and Q319 subjected to low-temperature stress for 24 h and their respective controls using Illumina HiSeq^TM^ 4000 high-throughput sequencing technology. Additionally, high-throughput metabolite sequencing was conducted on the samples using widely targeted metabolome sequencing technology. The results indicated that low-temperature stress triggered the accumulation of stress-related metabolites such as amino acids and their derivatives, lipids, phenolic acids, organic acids, flavonoids, lignin, coumarins, and alkaloids, suggesting their significant roles in the response to low temperature. This stress also promoted gene expression and metabolite accumulation involved in the flavonoid biosynthesis pathway. Notably, there were marked differences in gene expression and metabolites related to the glyoxylate and dicarboxylate metabolism pathways between B144 and Q319. This study, through multi-omics integrated analysis, provides valuable insights into the identification of metabolites, elucidation of metabolic pathways, and the biochemical and genetic basis of plant responses to stress, particularly under low-temperature conditions.

## 1. Introduction

Maize (*Zea mays* L.) is one of the most important food crops in the world, with its output accounting for 40% of global food production [1]. Maize is very sensitive to temperature during its growth and development period, with a maximum growth temperature of 25–28 °C and a minimum growth temperature of 5–18 °C. A temperature drop of 1 °C will delay the maturity period by 10 days, resulting in a reduction in production of more than 10% [2]. The northern spring maize region is a dominant area for maize production in China. In 2022, the maize planting area in this region was 16.45 million ha, accounting for 40.95% of the national maize planting area. The total output of maize was 121.24 million tons, accounting for 46.90% of the national total output of maize. It plays an important role in ensuring the stable and safe supply of national grain. However, due to special geographical reasons, low temperatures frequently occur in the northern spring maize region, which severely restricts the yield and quality of maize in this region.

The low-temperature tolerance of maize is a complex quantitative trait. Recent advances in omics have enabled us to decipher complex regulatory networks about plants’ stress resistance, specifically low-temperature tolerance [3,4,5,6] in the metabolome. Transcriptomics is a discipline that studies the transcription of genes and the regulation of transcription in cells at a holistic level and can quickly predict stress-related defense factors. Zeng et al. discovered that the natural variations of the *ZmRR1* gene exhibit a significant correlation with the low-temperature tolerance of maize inbred lines. *ZmRR1* InDel-35 encodes a protein containing a phosphorylation residue of mitogen-activated protein kinase (MPK). *ZmMPK8* negatively regulates low-temperature tolerance and interacts with *ZmRR1*, phosphorylating it at Ser15. The 45-bp deletion mutation encompassing Ser15 in *ZmRR1* prevents the phosphorylation of *ZmRR1* by *ZmMPK8*, thereby inhibiting the degradation of the *ZmRR1* protein mediated by the 26S protease pathway. Simultaneously, transcriptome analysis reveals that *ZmRR1* positively regulates the expression of the *ZmDREB1* and cellulose synthase (CesA) genes to enhance the low-temperature tolerance of maize [7]. Li et al. cloned the *ZmNAC3* gene in maize, which encodes a nuclear-targeted protein with a highly conserved NAC domain at its N-terminus. Several cis-acting elements in the promoter region of this gene that respond to stress were found, and the expression of the *ZmNAC3* gene is significantly up-regulated under high salinity and low-temperature conditions. The NAC-domain protein encoded by *ZmNAC3* may play a role in maize’s response to abiotic stress [8]. Jiang et al. used high-resolution mass spectrometry to analyze the non-target metabolomics of 245 maize inbred lines under normal and low-temperature treatments and, combined with mGWAS analysis, found that the transcription factor *ZmICE1* was involved in the metabolic regulation process induced by low temperatures. Overexpression of *ZmICE1* can significantly enhance the low-temperature tolerance of maize during germination and seedling stages, indicating that *ZmICE1* is a positive regulatory factor for maize low-temperature tolerance. Candidate gene association analysis identified SNP-465 in the *ZmICE1* promoter as an important functional site that affects the transcriptional regulation of *ZmICE1* and maize low-temperature tolerance. Variation at this site affects the binding of the *ZmMYB39* transcription factor to the *ZmICE1* promoter, thereby affecting the transcriptional level of *ZmICE1*, further clarifying that *ZmICE1* not only directly regulates the expression of the *DREB1* gene but also regulates the molecular mechanism of maize response to low temperature through regulating amino acid metabolism and reactive oxygen species levels [9]. Ma et al. identified the bZIP transcription factor gene *ZmbZIP4* in maize. The study found that *ZmbZIP4* was expressed to varying degrees in various organs of maize and was induced in seedlings treated with high salinity, drought, high temperature, low temperature, and abscisic acid. The transcription activation assay showed that *ZmbZIP4* had the function of a transcriptional activator; through immunoprecipitation sequencing technology, a genome-wide screening of *ZmbZIP4* targets was conducted, revealing that *ZmbZIP4* can positively regulate some stress response genes, gibberellin synthesis-related genes, and root development-related genes. Overexpressing *ZmbZIP4* can increase the synthesis of gibberellic acid, thereby improving the ability of plants to resist abiotic stress [10].

Metabolites are the final products of gene transcription and protein modification. Metabolomics is a science that qualitatively and quantitatively analyzes all metabolites with a molecular weight of less than 1000 in a certain organism, tissue, or cell [11]. The changes in metabolites under temperature stress are the result of the combined effects of genes and environment and are a direct reflection of the biochemical level and physiological phenotype of the organism. Yu et al. conducted a seedling low-temperature stress test on tolerance line B144 and susceptibility line Q319 and used targeted metabolomics methods to investigate their metabolite accumulation. The study found that the two lines accumulated different metabolites in response to low-temperature stress, among which nine metabolites, including guanosine 30, 50-cyclic monophosphate, sophoricoside-7-O-glucoside, L-lysine, L-phenylalanine, L-glutamine, kaempferol, and feruloyl tartaric acid, were clearly regulated in B144 and may become important cold-tolerant metabolites [12]. Urrutia et al. used metabolomics to determine the characteristics of metabolite in leaves at different temperatures. The study showed that some metabolites and proteins exhibited similar changes during all temperature decreases, among which the content of seven metabolites—trans-aconitate, coumaroyl-hydroxycitrate, geraniol glucoside rhamnoside, caffeoylquinic acid ester, ferrous quinic acid ester, isovitexin, and DIBOA-glucoside—showed a similar trend in early sowing field trials [13]. Duran Garzon et al. used the cold-sensitive line and cold-tolerant line of double haploid with significant differences in low-temperature tolerance to determine the main determinants of long-term low-temperature tolerance. The two lines were set to grow for 60 days under the conditions of 14 °C during the day and 10 °C at night. The study found that there was almost no change in the cell wall composition of the two genotypes. The low-temperature tolerance of cold-tolerant lines was related to higher chlorophyll content, glucose-6-phosphate dehydrogenase activity, and higher sucrose–starch ratio. The results showed that the main determining factor in low-temperature stress was starch–sucrose metabolism, and its regulatory effect affected the ability of cold-tolerant lines to cope with low-temperature stress [14].

Biological processes are complex and holistic, and transcriptomics can reveal differential gene expression and complex regulatory networks under different conditions, but it is difficult to accurately identify key genes and pathways. Metabolomics can analyze the changes in organisms affected by genetic or environmental factors, but due to the vast and complex metabolites in plants, metabolomics data cannot fully represent the entire plant metabolome. Therefore, integrating multiple omics can achieve a more comprehensive understanding of the “cause” to “effect” relationship and provide a deeper insight into the metabolic processes of plant low-temperature tolerance. This can help to comprehensively and systematically analyze the information transfer process and functional formation mechanism. Guo et al. studied the response mechanism of maize under low-temperature stress through transcriptome and metabolome analysis and found that the content of endogenous ABA increased under low-temperature stress, indicating that ABA plays a key role in plant adaptation to cold [15]. Yang et al. used transcriptome and metabolome techniques to study the response mechanism of broad beans to low-temperature stress. The results showed that the expression levels of multiple genes involved in lipid, amino acid, and lipid metabolism, as well as the accumulation of metabolites, increased during low-temperature stress. In addition, the expression levels of abscisic acid, gibberellin, and jasmonic acid were significantly different between cold-tolerant and cold-sensitive varieties. Flavonoids, methionine, and malondialdehyde can be used as biomarkers for plant chilling injury, indicating that these substances are related to plant responses to low-temperature stress [16]. Feng et al. compared the differences in response between waxy maize N28 and N67 under low-temperature stress and found that the expression levels of genes related to plant hormones and MAPK signaling pathways were significantly up-regulated in N28, and flavonoid metabolites were also significantly enriched in N28, indicating that these genes and metabolites play a key role in plant low-temperature tolerance, and may become potential target genes for breeding cold-resistant waxy maize [17]. After in-depth exploration by Xu et al., 6905 differentially expressed genes were identified in tobacco under low-temperature stress, mainly involved in signal transduction, carbohydrate metabolism, and phenylpropanoid biosynthesis. In addition, 35 protective metabolites were detected to participate in the cold stress response process, such as amino acids, carbohydrates, intermediates of the tricarboxylic acid cycle, and phenylpropanoid-related substances [18]. Li et al. compared the transcriptome and metabolome of pumpkin inbred lines under different temperature conditions, and the analysis showed that cold stress led to a significant increase in malondialdehyde content, relative conductivity, soluble protein, sugar content, and catalase activity. In addition, the transcription of different genes expressed in the plant hormone signaling pathway is also activated, and the transcription factor families such as AP2/ERF, bHLH, WRKY, MYB, and HSF are also activated [19].

The response of maize to low-temperature stress is the common result of the expression of multiple genes involving the expression of multiple genes and having a complex regulatory network. Despite some breakthroughs in understanding the genetic mechanisms behind low-temperature tolerance, low-temperature tolerance mechanisms remain largely unclear in maize regarding the transcriptome and metabolome. In an associated study, we screened a large set of maize inbred lines and identified two lines, Q319 and B144, that are sensitive and insensitive to low-temperature stress, respectively. Therefore, this study integrated transcriptome and metabolome to screen the differential genes and differential metabolites of two contrasting maize lines in response to low-temperature stress and explore the metabolic pathways and candidate genes of maize in response to low-temperature stress. This study provides new insights for the analysis of the molecular mechanism of plant low-temperature tolerance, which will lay a theoretical foundation for breeding new varieties of low-temperature tolerance.

## 2. Results

### 2.1. Principal Component Analysis (PCA)

**Figure 1:**
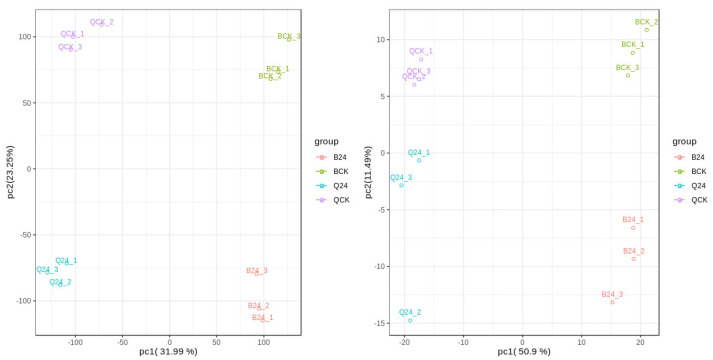
PCA score of differential genes and differential metabolites in response to low temperature.

By conducting a principal component analysis (PCA) on the genes and metabolites that respond to low-temperature differences, as shown in Figure 1, it can be seen that the samples are clustered together with minimal dispersion. The 12 samples are then divided into four groups by the principal component PCA1 (31.99%) of differential genes and the principal component PCA1 (50.90%) of differential metabolites. PCA2 clearly separates the two materials, indicating that after low-temperature stress treatment, different materials respond to low-temperature stress with significant differences. The three biological replicates of the same material sample have small differences, indicating high sample reproducibility. The differences between different materials and treatment samples are significant, allowing for subsequent analysis of the results.

### 2.2. Correlation Analysis

**Figure 2:**
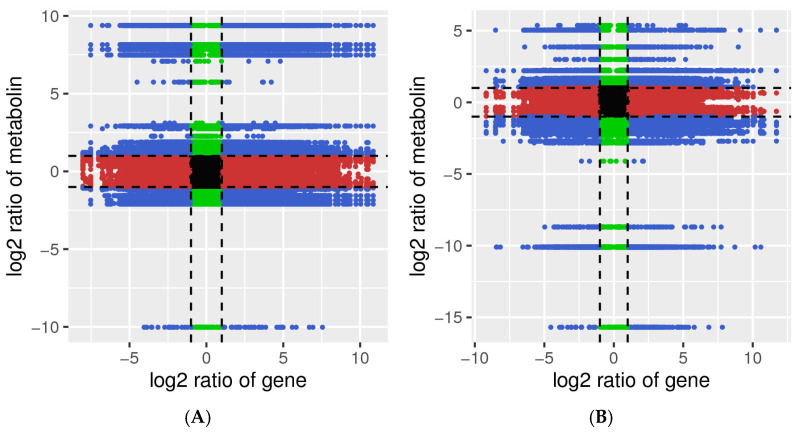
Nine-quadrant diagram of the correlation between differential metabolites and differential genes. Note: (**A**) Nine-quadrant diagram of MBCK vs. MB24 and TBCK vs. TB24. (**B**) Nine-quadrant diagram of MQCK vs. MQ24 and TQCK vs. TQ24. T stands for transcriptome, and M stands for metabolome.

Correlation analysis was conducted on the detected low-temperature differential genes and differential metabolites. The Pearson correlation coefficient between genes and metabolites was calculated using the cor program in *R* language. The differential folds of genes and metabolites with Pearson correlation coefficients greater than 0.8 in each differential group were displayed using a nine-quadrant diagram, as shown in Figure 2. During germination at low temperatures, the B144 had 22,691 differential genes and 60 corresponding differential metabolites in the third and seventh quadrants. During the germination of the Q319 at low temperatures, there were 17,367 differentially expressed genes and 64 corresponding differentially expressed metabolites in the third and seventh quadrants.

### 2.3. KEGG Enrichment and Pathway Analysis

**Figure 3:**
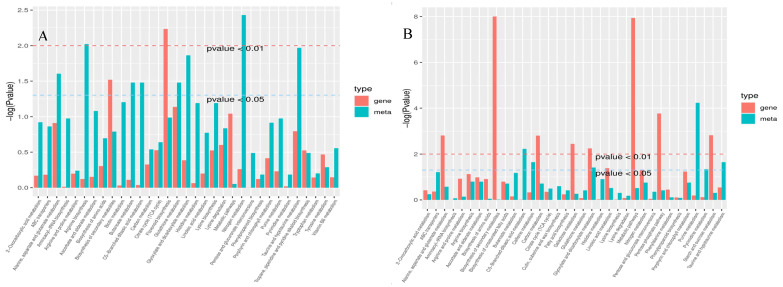
*p*-value histogram of enrichment analysis of differential gene and differential metabolite. Note: The horizontal axis in the KEGG enrichment diagram represents metabolic pathways, and the red color in the vertical axis represents the enrichment *p*-value of differential genes, while the green color represents the enrichment *p*-value of differential metabolites, represented by −log(*p*-value). The higher the vertical axis, the stronger the enrichment degree. (**A**) shows the B144 KEGG enrichment diagram, and (**B**) shows the Q319 KEGG enrichment diagram.

KEGG pathway enrichment analysis was conducted on differential genes and metabolites, and pathways with *p*-values < 0.05 in at least one omics datapoint were screened. As shown in Figure 3A and Table 1, the B144 was significantly enriched in 10 pathways, including 12 differential genes and three differential metabolites significantly enriched in arginine biosynthesis (ko00220); 26 differential genes and three differential metabolites significantly enriched in alanine, aspartate, and glutamate metabolism (ko00250); 4 differential genes and two differential metabolites significantly enriched in taurine and hypotaurine metabolism (ko00430); 48 differential genes and two differential metabolites significantly enriched in glutathione metabolism (ko00480); 31 differential genes and three differential metabolites significantly enriched in glyoxylate and dicarboxylate metabolism (ko00630); 8 differential genes and two differential metabolites significantly enriched in butanoate metabolism (ko00650); 2 differential genes and two differential metabolites significantly enriched in C5-branched dibasic acid metabolism (ko00660); 15 differential genes and two differential metabolites significantly enriched in nitrogen metabolism (ko00910); 22 differential genes and two differential metabolites significantly enriched in flavonoid biosynthesis (ko00941); and 500 differential genes and nine differential metabolites significantly enriched in biosynthesis of secondary metabolites (ko01110). As shown in Figure 3B and Table 2, the Q319 significantly enriched 13 pathways, including 34 differentially expressed genes and one differentially expressed metabolite that significantly enriched the pentose phosphate pathway (ko00030); 57 differentially expressed genes and one differentially expressed metabolite that significantly enriched starch and sucrose metabolism (ko00500); 29 differentially expressed genes and two differentially expressed metabolites that significantly enriched alanine, aspartate, and glutamate metabolism (ko00250); 110 differentially expressed genes and three differentially expressed metabolites that significantly enriched carbon metabolism (ko01200); 30 differentially expressed genes and one differentially expressed metabolite that significantly enriched galactose metabolism (ko00052); 8 differentially expressed genes and two differentially expressed metabolites that significantly enriched linoleic acid metabolism (ko00591); 18 differentially expressed genes and one differentially expressed metabolite that significantly enriched nitrogen metabolism (ko00910); 3 differentially expressed genes and two differentially expressed metabolites that significantly enriched taurine and hypotaurine metabolism (ko00430); 1 differentially expressed gene and two differentially expressed metabolites that significantly enriched caffeine metabolism (ko00232); 17 differentially expressed genes and four differentially expressed metabolites that significantly enriched pyrimidine metabolism (ko00240); 30 differentially expressed genes and eight differentially expressed metabolites that significantly enriched purine metabolism (ko00230); 1 differentially expressed gene and three differentially expressed metabolites that significantly enriched C5-branched dibasic acid metabolism (ko00660); and 36 differentially expressed genes and three differentially expressed metabolites that significantly enriched glyoxylate and dicarboxylate metabolism (ko00630).

B144 and Q319 were enriched in five pathways, including alanine, aspartate, and glutamate metabolism (ko00250); taurine and hypotaurine metabolism (ko00430); glyoxylate and dicarboxylate metabolism (ko00630); C5-branched dibasic acid metabolism (ko00660); and nitrogen metabolism (ko00910), where the *p*-value_gene and *p*-value_meta of glyoxylate and dicarboxylate metabolism (ko00630, Figure S1) in Q319 were 0.006 and 0.039, respectively, indicating that this pathway was significantly enriched in the transcriptome and metabolome of Q319.

### 2.4. Cluster Analysis and Correlation Network Analysis

**Figure 4:**
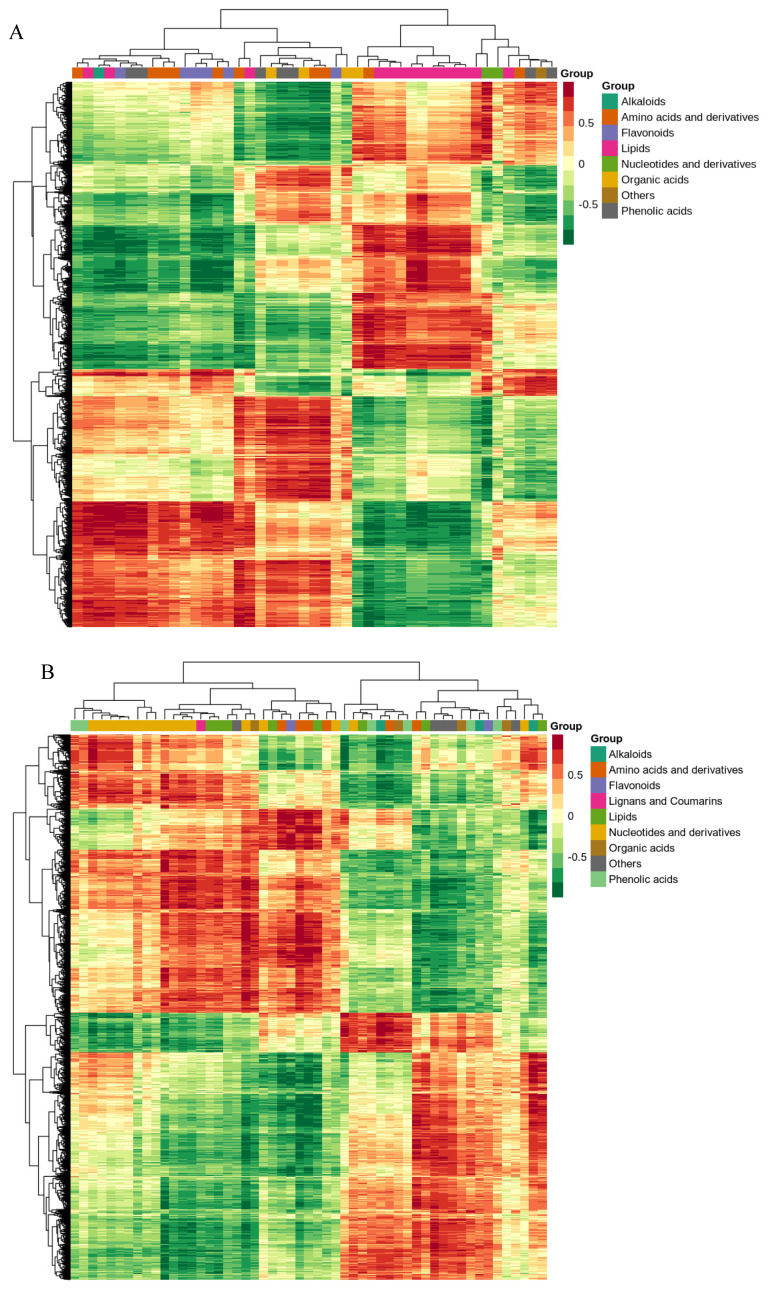
Cluster analysis of differential genes and differential metabolites. Note: (**A**) shows the differential expression gene and differential metabolite cluster heatmap after B144 low-temperature stress, and (**B**) shows the differential expression gene and differential metabolite cluster heatmap after Q319 low-temperature stress.

In the above correlation analysis, for differential genes and differential metabolites with a correlation coefficient of 0.8 or higher, all correlation calculation results were selected to draw a correlation coefficient clustering heatmap, representing the correlation between metabolites and genes through a network diagram. Differential genes and differential metabolites with a correlation coefficient greater than 0.8 in each pathway were selected for plotting. From Figure 4A,B, it can be seen that the differential genes and metabolites produced by B144 and Q319 under low-temperature stress mainly cluster in lipids, organic acids, amino acids and derivatives, flavonoids, phenolic acids, nucleotides and derivatives, lignans and coumarins, alkaloids, and others.

**Figure 5:**
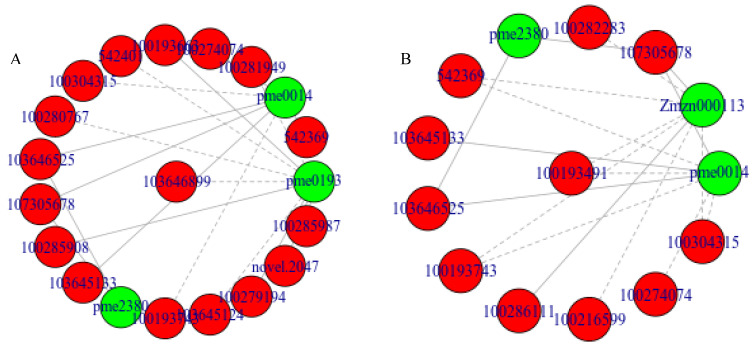
Correlation network analysis of differential genes and differential metabolites. Note: (**A**) shows the network diagram of differentially expressed genes and differentially metabolized glyoxylate and dicarboxylate metabolism (ko00630) after B144 low-temperature stress. (**B**) shows the network diagram of differentially expressed genes and metabolized glyoxylate and dicarboxylate metabolism (ko00630) after Q319 low-temperature stress. Metabolites are marked in green, and genes are marked in red. Solid lines represent positive correlation, and dashed lines represent negative correlation.

Based on the analysis of the differentially expressed genes and the differentially metabolized pathway glyoxylate and dicarboxylate metabolism (ko00630) in B144 and Q319 under low-temperature stress, as shown in Figure 5A,B, it was found that there are 19 differentially expressed genes corresponding to three differentially metabolites in B144 in this pathway, of which 7 differentially expressed genes corresponded to the differential metabolite pme0014, which were 542369, 100274074, 100304315, 103646525, 107305678, 103645133, and 100193743. The 10 differential genes corresponding to the differential metabolite pme0193 are 103646899, 100281949, 100193663, 542401, 100280767, 100285908, 103645124, 100279194, novel.2047, and 100285987. The two differential genes corresponding to the differential metabolite pme2380 are 103,646,525 and 107305678, respectively. Q319 has enriched 18 differential genes corresponding to three differential metabolites in this pathway, of which 8 differential genes correspond to differential metabolite pme0014, which are 100193491, 542369, 100274074, 100304315, 103646525, 107305678, 103,645,133 and 100193743. The eight differential genes corresponding to the differential metabolite Zmzn000113 are 100193491, 100282283, 107305678, 542369, 100193743, 100286111, 100216599, and 100304315. The two differential genes corresponding to the differential metabolite pme2380 are 103,646,525 and 107305678, respectively. A gene can be associated with multiple metabolites, or a metabolite can be associated with multiple genes. The differential gene 107,305,678 in Q319 corresponds to three differential metabolites: pme0014, Zmzn000113, and pme2380.

### 2.5. O2PLS Analysis

**Figure 6:**
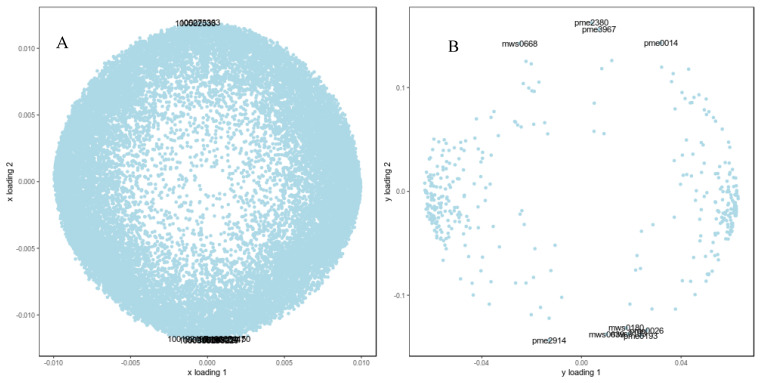
O2PLS model loading graph. Note: (**A**) represents the transcriptome loading plot, and (**B**) represents the metabolome loading plot.

In this study, we selected all the differential genes and metabolites measured during the germination process under low-temperature stress in B144 and Q319 to establish an O2PLS model. Through the loading plot, we initially identified variables with high correlation and weight in different data sets and screened out important variables that affect the other omics. As shown in Figure 6, the horizontal and vertical axes represent the load values of the variables under different experimental conditions. The closer the variable is to the diagonal, the smaller the difference between the two conditions. Variables that are far from the diagonal are significantly changed variables. The distance from each point to the origin means the size of the correlation with another omics. The top 10 genes and metabolites that have a greater impact on other omics are indicated in the figure. The top 10 genes are 100273363, 100285220, 103633615, 100502533, 109942247, 103654150, 100192497, 100281277, 100382514, and 100216746. The top 10 metabolites are α-ketoglutaric acid (pme2380), 2-(dimethylamino)guanosine (pme3967), L-glutamic acid (pme0014), xanthosine (mws0668), 3-hydroxy-3-methylpentane-1,5-dioic acid (pme2914), L-glutamine (pme0193), 3,4-dihydroxybenzoic acid (mws0183), 2,3-dihydroxybenzoic acid (mws0639), L-lysine (pme0026), and 2,5-dihydroxybenzoic acid (mws0180).

### 2.6. Analysis of Low-Temperature Tolerance Mechanism in Seedling Stage

This study combined transcriptomics and metabolomics to analyze the changes in differentially expressed genes, differential metabolites, and important metabolic pathways in response to low-temperature stress of B144 and Q319. In this study, we found that the differential genes and metabolites produced by B144 and Q319 under low-temperature stress were mainly clustered in organic acids, amino acids and their derivatives, lipids, phenolic acids, flavonoids, lignin, coumarins, alkaloids, and other substances. This indicates that the differences in low-temperature tolerance between B144 and Q319 are related to the synthesis and metabolism of these substances.

The results of the KEGG pathway enrichment analysis showed that the flavonoid metabolic pathway was significantly enriched in B144 and 22 differential genes, including cytochrome P450 superfamily protein, 4-monooxygenase of cinnamic acid, caffeoyl-CoA O-methyltransferase 1, chalcone synthase, hydroxycinnamoyltransferase, etc., which were significantly enriched, while this pathway was not found in Q319, indicating that B144 could enhance the tolerance to low-temperature stress through the up-regulation of the expression of flavonoid metabolites. In addition, the glyoxylic acid and dicarboxylic acid metabolism pathway was significantly enriched in Q319, with a *p*-value of 0.006 for differentially expressed genes; the network analysis of the metabolic pathway revealed that B144 and Q319 enriched the same differentially expressed metabolites in this metabolic pathway, namely L-glutamate and alpha-ketoglutaric acid, indicating that these two differentially expressed metabolites play an important role in the response to low-temperature stress.

### 2.7. Validation of Transcriptome by qPCR

**Figure 7:**
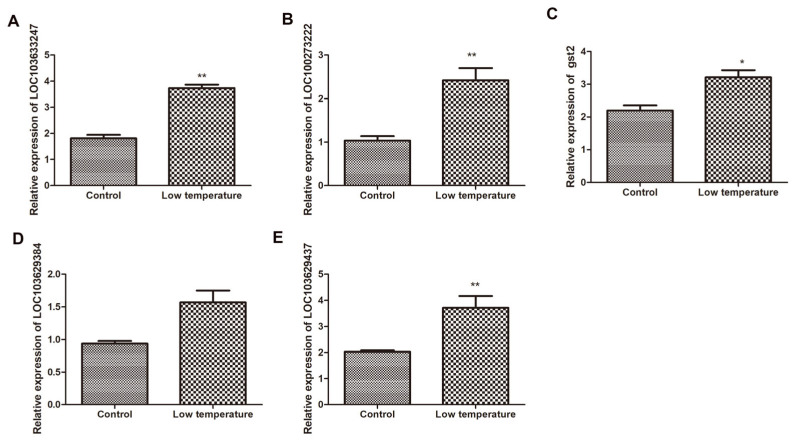
Detection of mRNA expression level of candidate genes in B144 by qPCR. Note: (**A**) represents the expression level of *LOC*103633247; (**B**) represents the expression level of *LOC*100273222; (**C**) represents the expression level of *gst2*; (**D**) represents the expression level of *LOC*103629384; (**E**) represents the expression level of *LOC*103629437. * and ** denote levels of significance at *p* < 0.05 and *p* < 0.001, respectively.

**Figure 8:**
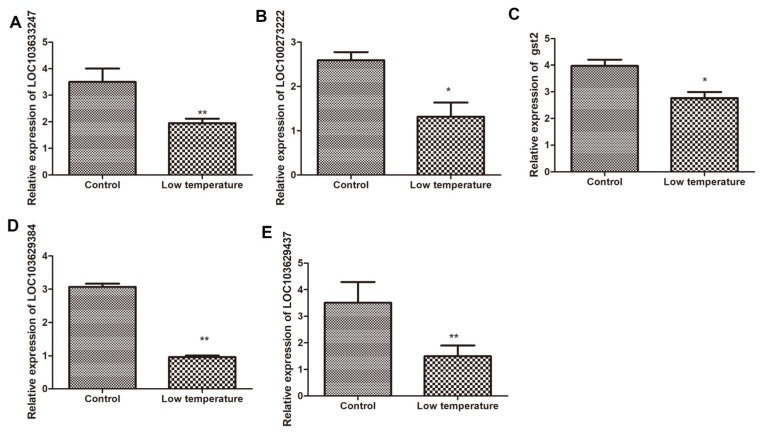
Detection of mRNA expression level of candidate genes in Q319 by qPCR. Note: (**A**) represents the expression level of *LOC*103633247; (**B**) represents the expression level of *LOC*100273222; (**C**) represents the expression level of *gst2*; (**D**) represents the expression level of *LOC*103629384; (**E**) represents the expression level of *LOC*103629437. * and ** denote levels of significance at *p* < 0.05 and *p* < 0.001, respectively.

We validated the expression of five candidate genes that were differentially expressed in response to low temperatures (Figure 7 and Figure 8). The maize *Actin1* gene was used as an internal control to standardize the data, and the number of four genes’ transcripts was normalized by comparing them with the constitutive abundance of *Actin1*. All the tested genes were characterized by similar expression in the transcriptome data.

## 3. Discussion

Changes in metabolic processes play an important role in plant responses to abiotic stress. Plants produce various metabolites through metabolic responses to cope with abiotic stress, helping them to cope with the constantly changing external environment [20]. The primary metabolites produced by plant stress metabolism, such as carbohydrates, lipids, organic acids, amino acids, and secondary metabolites, such as phenolic compounds and flavonoids, play a crucial role in plant growth and development, stress adaptation, and plant defense [21,22].

### 3.1. Amino Acids Metabolites Involved in Low-Temperature Stress

Research has shown that primary metabolites and secondary metabolites play an important role in plants’ responses to abiotic stress [23]. As a penetration protectant, amino acids can serve as substrates for the synthesis of antioxidant compounds under stress conditions, maintaining the redox homeostasis of cells [24]. Xu et al. used the transcriptome and metabolome to jointly analyze the specific expression of genes and metabolites in tobacco during cold adaptation. The results showed that on the LC-MS platform, 35 different metabolites had significant changes during cold adaptation, among which the accumulation of amino acids such as phenylalanine, tyrosine, and leucine in tobacco leaves significantly increased, indicating that tobacco improved its cold adaptation ability mainly by activating amino acid metabolism and synthesizing sugars, TCA cycle intermediates and phenolic compounds [18]. Yang et al. conducted metabolome analysis on common soybeans and salt-tolerant soybeans, and the results showed that salt-tolerant wild soybeans improved the metabolism of amino acids and organic acids in the body [25]. Our results showed that the contents of alanine, aspartate, arginine, taurine, and glutamic acid increased, and the accumulation of these amino acids helped to enhance the low-temperature tolerance of maize through mechanisms such as osmotic protection, antioxidant defense, energy metabolism, and reduction of oxidative stress damage.

### 3.2. Lipids Metabolites Involved in Low-Temperature Stress

Lipids also play a very important role in plant response to low-temperature stress [26]. ACKAH et al. studied the effects of drought stress on the metabolism of mulberry Yu-711 and found that the levels of total lipids, galactolipids, and phospholipids changed significantly, accounting for 48% of the total differentially expressed metabolites, indicating that mulberry responds to drought stress by regulating lipid metabolites [27]. Li et al. studied the effect of cold stress on the rice cultivar Zhonghua 11 microspores by integrated transcriptomics and metabolomics analysis and found that pathways related to amino acid and nucleotide metabolism were up-regulated, while pathways related to carbohydrate metabolism were down-regulated. Most of the differentially expressed metabolites belonged to lipids and lipid-like molecules [28]. Xu et al. conducted transcriptome and metabolome analyses on cold-sensitive and cold-tolerant highland barley varieties under low-temperature stress. Studies have found that the level of lipid content significantly affected the low-temperature tolerance of highland barley. In addition, C-repeat CRT binding factor 10AHvCBF10A and lipase HvGDSL were key genes that actively regulate lipid metabolism to improve the low-temperature tolerance of bacterial wilt [29]. Jian et al. conducted transcriptome and metabolome analyses on leaf samples of five spring rapeseed ecotypes and five winter rapeseed ecotypes at 4 °C and 8 °C. After cold stress, 25,460 differentially expressed genes (DEGs) and one differentially expressed metabolite (DEMs) were identified in the spring rapeseed ecotype, and 28,512 DEGs and 47 DEMs were identified in the winter rapeseed ecotype. The differences in gene expression and metabolite levels after cold stress treatment are one of the reasons for the low-temperature tolerance of different rapeseed ecotypes. The results suggest that lipid metabolism, signal transduction, and transcription factors may play different roles in the response of spring and winter ecotype rapeseed to cold stress [30].

### 3.3. Phenolic Metabolites Involved in Low-Temperature Stress

Phenolic compounds are secondary metabolites containing phenolic hydroxyl groups, which play an important role in plant defense against abiotic stress [31,32]. Under water stress, compared with stress-sensitive genotypes, the total phenolic acid content in the leaf of stress-tolerant genotypes of durum wheat is higher, and the total phenolic acid content in mature grains is higher compared with the control [33]. Research indicates that when wheat and corn are subjected to salt stress, the content of total phenolic and flavonoid compounds increases [34,35]. Under heat stress, the accumulation of soluble phenolic substances in tomato and watermelon plants significantly increases [36]. Flavonoids are a class of important polyphenolic compounds that have various biological functions in plants. In this study, under low-temperature stress, the expression levels of flavonoid biosynthesis pathway-related genes and intermediate metabolites were up-regulated in B144, indicating that phenolic metabolism plays a key role in maize’s resistance to low-temperature stress. Through cluster analysis, we found that the differential genes and metabolites produced by B144 and Q319 under low-temperature stress were mainly clustered in organic acids, amino acids and their derivatives, lipids, phenolic acids, flavonoids, lignin, coumarins, alkaloids, and other substances. This indicates that the differences in low-temperature tolerance between B144 and Q319 are related to the synthesis and metabolism of these substances.

The results of KEGG pathway enrichment analysis in this study showed that the low-temperature-tolerant maize inbred line B144 was significantly enriched in 10 pathways, while the low-temperature-sensitive maize inbred line Q319 was significantly enriched in 13 pathways. The flavonoid metabolic pathway was significantly enriched in B144, with a *p*-value of 0.006 for differentially expressed genes, while this pathway was not found in Q319. In addition, B144 and Q319 were enriched in five pathways, including alanine, aspartate and glutamate metabolism, taurine and low taurine metabolism, glyoxylic acid and dicarboxylic acid metabolism, C5-branched dicarboxylic acid metabolism, and nitrogen metabolism. Among them, the glyoxylic acid and dicarboxylic acid metabolism pathway was significantly enriched in Q319, with a *p*-value of 0.006 for differentially expressed genes. However, the *p*-value for differentially expressed genes in B144 was 0.917, indicating a significant difference in gene expression, suggesting that flavonoid metabolism pathways and glyoxylic acid and dicarboxylic acid metabolism pathways may be related to the differences in low-temperature tolerance between these two lines.

### 3.4. Flavonoid Metabolites in Response to Low-Temperature Stress

As an important secondary metabolite, flavonoid metabolites play an important role in plant abiotic stress resistance. The increase in flavonoid content helps plants effectively resist low-temperature stress [37,38]. Flavonoids not only have the ability to scavenge ROS but also serve as signaling molecules to activate defense-related signaling pathways and regulatory mechanisms [39]. Research has shown that low-temperature stress enhances the accumulation of flavonoids in *Arabidopsis thaliana* leaves, and flavonoids are a decisive factor in the freezing resistance and cold adaptation of *Arabidopsis thaliana* [40]. Yu et al. conducted a joint analysis of transcriptome and metabolome in mango leaves and detected a total of 1323 metabolites. Among them, flavonoids, amino acids and their derivatives, and lipids in golden leaves accumulated more after low-temperature stress, indicating that the low-temperature tolerance of mango is related to the accumulation of flavonoids [41]. Low temperature induces the significant up-regulation of enzyme genes encoding flavonoid biosynthesis such as CHS, F3H, and DFR in *Arabidopsis thaliana* and *Ginkgo biloba* leaves, purple cabbage, and grape skin, enhancing the low-temperature tolerance of plants [42,43,44,45]. Previous studies have shown that flavonoids such as flavonols, flavanols, and anthocyanins have strong cold resistance. Wu et al. conducted transcriptome and metabolome analysis on *Dendrobium huoshanensis* and obtained a total of 23,724 DEGs. Among them, 12 DEGs are related to polysaccharides, flavonoids, and hormone pathways. Pathway analysis showed that “flavonoid biosynthesis”, “anthocyanin biosynthesis”, and “flavonoid and flavonol biosynthesis” play an important role in the response of *Dendrobium huoshanensis* to cold stress [46]. The above research results further confirm the important role of flavonoids in enhancing cold resistance. Our research results found that compared with Q319, the differential genes in B144 were significantly enriched in the flavonoid compound metabolism pathway, including 22 differential genes, namely cytochrome P450 superfamily protein, 4-monooxygenase of cinnamic acid, caffeoyl-CoA O-methyltransferase 1, chalcone synthase, hydroxycinnamoyltransferase, etc., indicating that B144 can up-regulate the expression of flavonoid metabolites, thereby improving the tolerance to low-temperature stress.

### 3.5. Glyoxylate and Dicarboxylate Signal Pathway in Response to Low-Temperature Stress

The glyoxylate and dicarboxylic acid cycle metabolism mainly participates in the conversion of fat to sugar in plants, and many products produced during metabolism have been shown to have the function of improving plant stress resistance [47]. Huang et al. used transcriptomics, proteomics, and metabolomics to analyze the response mechanism of marine red algae to intertidal drying stress. The results showed that multiple pairs of differential genes, differential proteins, and differential metabolites were involved in glyoxylic acid and dicarboxylic acid metabolism and responded to stress through osmoregulation and scavenging of ROS [48]. Cheng et al. used RNA-seq to identify key genes and metabolic pathways associated with low-temperature tolerance in Helictotrichon virescens. The results showed that the expression levels of three central gene clusters were significantly higher than those under control conditions. At the same time, these three central genes were mainly enriched in metabolic pathways such as sphingolipids, glyoxylate and dicarboxylate, and carotenoid biosynthesis [49]. In this study, the network analysis of glyoxylic acid and dicarboxylic acid metabolic pathways found that B144 was enriched in 19 differentially expressed genes and three differentially expressed metabolites in this metabolic pathway. These differentially expressed genes were catalase, aconitate hydrolase 3, acetyl-CoA synthase, malate dehydrogenase, serine-glyoxylate aminotransferase, malonate dehydrogenase, etc. The differentially expressed metabolites were L-glutamic acid (pme0014), L-glutamine (pme0193), and α-ketoglutarate (pme2380); Q319 enriched a total of 18 differentially expressed genes and three differentially expressed metabolites in this pathway. These differentially expressed genes were aconitate hydrolase 3, acetyl-CoA synthase, malate dehydrogenase, serine-glyoxylate aminotransferase, acetyl-CoA acetyltransferase, formyl tetrahydrofolic acid dehydratase, etc. The differentially expressed metabolites were L-glutamic acid (pme0014), L-threonine-3-methylaspartate (Zmzn000113), and α-ketoglutarate (pme2380). The results showed that both B144 and Q319 contained the same differentially expressed metabolites, namely L-glutamic acid (pme0014) and α-ketoglutaric acid (pme2380). One metabolite can be associated with multiple genes; for example, there are seven differentially expressed genes and eight differentially expressed genes in B144 and Q319, respectively, corresponding to the same differential metabolite L-glutamic acid (pme0014). In B144 and Q319, there are two differentially expressed genes corresponding to the same differential metabolite α-ketoglutaric acid (pme2380), indicating that L-glutamic acid and α-ketoglutaric acid play an important role in the response to low-temperature stress. Previous studies have reported that L-glutamic acid is a multifunctional amino acid that occupies a central position in amino acid metabolism and is also a precursor of glutamine, proline, and arginine [50]. L-glutamic acid is considered to be an important regulatory factor for plant growth and development under low-temperature stress [51,52]. Hou et al. studied the freezing damage of prunus domestica fruits under freezing stress treated with L-glutamic acid and found that L-glutamic acid can significantly increase the expression of PdGLRs, the concentration of cytoplasmic Ca^2+^, the content of CaM and CML, reduce the accumulation of ROS, increase the content of GABA and energy levels, and thus improve the antioxidant capacity of prunus domestica fruits, which helps to maintain the integrity of the mitochondrial structure of frozen prunus domestica fruits [53]. Li et al. reported that in tomatoes, pretreatment with L-glutamic acid under low-temperature stress can up-regulate the expression of GLR3.3/GLR3.5, activate Ca^2+^ signaling, and thereby increase the resistance of tomato plants to low-temperature stress [54]. Zheng et al. have demonstrated that two members of the Arabidopsis glutamate-like receptor family, AtGLR1.2 and AtGLR1.3, play a positive role in the response of plants to cold stress. AtGLR1.2 and AtGLR1.3 promote the downstream CBF/DREB1 cold response pathway during cold stress by activating endogenous jasmonic acid accumulation, thereby improving the low-temperature tolerance of *Arabidopsis thaliana* [55]. α-ketoglutarate plays a bridge role in the metabolism of carbohydrates and amino acids. It is not only a precursor for the biosynthesis of glutamic acid and proline but also participates in the subsequent protein biosynthesis. The role of α-ketoglutarate in low-temperature tolerance is related to enhancing the function of antioxidant systems and promoting the synthesis of amino acids [56]. In addition, the differential gene malate dehydrogenase in Q319 corresponds to three differential metabolites, pme0014, Zmzn000113, and pme2380, indicating that a gene can be associated with multiple metabolites. Our research results show that glutamate, α-ketoglutarate, acetyl-CoA synthase, malate dehydrogenase, and serine-glyoxylate aminotransferase have been identified in both B144 and Q319, which means that they play a key role in the glyoxylate and dicarboxylic acid metabolic pathways. These results further confirm the role of glyoxylic acid and dicarboxylic acid metabolism in regulating plant growth, development, defense responses, and signaling in response to low-temperature stress.

## 4. Materials and Methods

### 4.1. Plant Materials

The seeds of two inbred lines, B144 (tolerant) and Q319 (sensitive), were provided by the Maize Research Institute of Heilongjiang Academy of Agricultural Sciences, China. B144, a highly low-temperature-tolerant inbred line, is the female parent of the large-scale corn hybrid Jidan 27 in Northeast China, while Q319, a line that is highly susceptible to low temperatures, is selected from the American hybrid 78,599 [2]. The seed surfaces of both inbred lines were sterilized with 0.5% sodium hypochlorite solution, washed with distilled water, and germinated in pots for 10 days at 25 °C in a 12/12 h photoperiod in a light incubator (GEN1000, Conviron, Pembina, ND, USA), with five replicates of ten plants per treatment. Temperature was then set to 5 °C to induce a low-temperature stress response, while under control conditions, the temperature was kept constant. A group of seedlings continued to grow at normal conditions to be used as a control. The leaf samples were collected after 24 h from both inbred lines in three biological and technical repeats. A total of 12 samples were stored at −80 °C.

### 4.2. Transcriptome Sequencing Analysis

Total RNA of each sample was extracted using TRIzol reagent (Invitrogen) following manufacturer’s recommendations. RNA purity, concentration, and integrity were checked by using the NanoPhotometer^®^spectrophotometer (IMPLEN, Westlake Village, CA, USA) and 1% agarose gel electrophoresis. The mRNA was purified from total RNA with poly-T oligo-attached magnetic beads. The cleaved RNA fragments were reverse-transcribed to double-strand cDNA using random hexamer primer. The cDNA fragments were purified with AMPure XP system (Beckman Coulter, Beverly, CA, USA), blunted with phosphate at 5′end and stickiness “A” at 3′end, and adaptor-ligated. These products were subsequently purified (AMPure XP system) and amplified by PCR with Phusion High-Fidelity DNA Polymerase, Universal PCR Primers, and Index (X) Primer to create cDNA libraries. Finally, the cDNA libraries were sequenced using Illumina HiseqTM 4000 at Nuohe Zhiyuan Company, Ltd. (Beijing, China; https://en.novogene.com/; accessed on 28 September 2020). Raw data of Fastq format were processed through in-house Perl scripts to obtain clean data. The clean data were compared with the maize reference genome (B73 RefGen-v4) by using HIsat2 v2.0.5 software [57]. FeatureCounts software (v.2.0.3) was used to calculate the Fragments Per Kilobase of transcript per Million fragments mapped (FPKM) value of each gene. The DESeq method was used to calculate differentially expressed genes (DEGs) [58]. DEGs with False Discovery Rate (FDR) < 0.01 and log2 FC ≥ 1 and ≤−1 were considered for further analyses.

### 4.3. Sample Preparation and Metabolic Profiling Analysis

The samples were freeze-dried in a vacuum and then ground to powder. The powder (100 mg) was weighed, followed by thawing in 1.2 mL methanol extract (Shanghai Aladdin Bio-Chem Technology Co., Ltd., Shanghai, China). After cyclic centrifugation (30 s after every 30 min), the samples were kept at 4 °C overnight. The supernatant was collected by centrifugation at 12,000 rpm for 10 min and filtered by 0.22 μm filters for LC-MS analysis [59].

In this study, metabolome was quantified using ultra-performance liquid chromatography–tandem mass spectrometry (UPLC-MS/MS; SHIMADZU NexeraX2, www.shimadzu.com; accessed on 11 November 2021; Applied Biosystems 4500 Q TRAP, www.appliedbiosystems.com; accessed on 11 November 2021) on a UPLCMS/MS system (Metware, Wuhan, China). The analytical conditions were as follows: UPLC: column; Agilent SB-C18 (1.8 μm × 2.1 mm × 100 mm) was used while the mobile phase with A (pure water with formic acid) and B (acetonitrile with formic acid) solvents was standardized. The gradient program was used starting from 95% A and 5% B for 9 min, then changed to 5% A and 95% B for 1 min, and finally adjusted back to 95% A and 5% B. The flow rate was 0.35 mL/min throughout the gradient process with a column temperature of 40 °C and 4 µL injection volume. The effluent was connected to an ESI-triple quadrupole-linear ion trap mass spectrometer (AB4500 Q TRAP UPLC/MS/MS) [60]. Analyst 1.6.3 was used to control the system parameters [61]. The ESI source operation parameters were kept as explained by [62]. The differential accumulation of metabolites (DAMs) between B144 and Q319 under low-temperature stress compared with the control was screened for variable importance in projection (VIP) ≥ 1 and log2FC ≥ 1.

### 4.4. Integration Analysis of Transcriptome and Metabolome

The “prcomp” package in *R* software (www.r-project.org/; accessed on 27 November 2020) was used to perform principal component analysis (PCA) on samples for comparison. The Pearson correlation coefficients (PCC) of DEGs and DAMs in each differential grouping were calculated using the cor function in *R*. The results with correlation coefficients greater than 0.8 were selected for correlation coefficient clustering heat map and correlation network diagram analysis. Data from the nine-quadrant plot were processed by integrating fold changes with correlation coefficients and visualized using the points function in *R*. The two-way orthogonal partial least squares (O2PLS) model was employed to integrate and evaluate the overall correlation of the metabolomic and transcriptomic data.

### 4.5. Transcriptome Data Validation by qPCR

Five candidate genes that were differentially expressed in response to low temperature were selected for qPCR to verify the reliability of the transcriptome data. The primers were designed in Beacon Designer software (version 7.8; http://www.premierbiosoft.com/molecular_beacons/; accessed on 8 November 2010 ) (Table 3). The maize *Actin1* gene (GenBank Accession Number: GRMZM2G126010) was used as an internal control. qPCR was performed using the ABI7500 Real-Time System (Applied Biosystems) according to the instructions of SYBR^®^ Green Real-time PCR Master Mix (TOYOBO, Japan). Reaction conditions were 95 °C for 30 s, followed by 40 cycles of denaturation at 95 °C for 5 s, annealing at 60 °C for 30 s, and extension at 72 °C 30 s. The relative expression of genes was calculated by 2-ΔΔCT, and variance and significance were analyzed by SPSS Statistics v23.0 software (IBM, New York, NY, USA).

## 5. Conclusions

This study comprehensively analyzed the changes in the abundance of transcription genes and metabolites during the response of maize to low-temperature stress and compared the transcriptome and metabolome changes of B144 and Q319 under low-temperature stress. The results showed that low-temperature stress widely activated cold signaling pathways and triggered the accumulation of a large number of stress-related metabolites, including amino acids and their derivatives, lipids, phenolic acids, organic acids, flavonoids, lignin, coumarins, and alkaloids, laying the foundation for the metabolic changes in low-temperature tolerance in maize. The comprehensive analysis of the gene-metabolite network showed that low-temperature stress promoted the expression of genes involved in the flavonoid biosynthesis pathway and the accumulation of metabolites, indicating that these secondary metabolites play an important role in stress tolerance. In addition, the gene expression and metabolites involved in the glyoxylic acid and dicarboxylic acid metabolic pathways were significantly different between B144 and Q319, indicating that these genes and metabolites may be potential target genes for breeding cold-resistant maize, but further research is needed. The integration of metabolomics and transcriptomics provides important reference for the identification of plant metabolites under stress, the analysis of metabolic pathways, and the biochemical and genetic basis of plant response to stress.

## Figures and Tables

**Table 1:** Number of differentially expressed genes and metabolites significantly enriched in KEGG pathways in B144.

KO-Id	Kegg_Pathway	Gene Count	Meta Count	*p*-Value Gene	*p*-Value Meta
ko00941	Flavonoid biosynthesis	22	2	0.006	0.103
ko01110	Biosynthesis of secondary metabolites	500	9	0.030	0.162
ko00480	Glutathione metabolism	48	2	0.073	0.033
ko00250	Alanine, aspartate, and glutamate metabolism	26	3	0.123	0.025
ko00430	Taurine and hypotaurine metabolism	4	2	0.161	0.011
ko00630	Glyoxylate and dicarboxylate metabolism	31	3	0.413	0.014
ko00910	Nitrogen metabolism	15	2	0.549	0.004
ko00220	Arginine biosynthesis	12	3	0.758	0.010
ko00650	Butanoate metabolism	8	2	0.775	0.033
ko00660	C5-branched dibasic acid metabolism	2	2	0.917	0.033

**Table 2:** Number of differentially expressed genes and metabolites significantly enriched in KEGG pathways in Q319.

KO-Id	Kegg_Pathway	Gene Count	Meta Count	*p*-Value Gene	*p*-Value Meta
ko00030	Pentose phosphate pathway	34	1	1.685 × 10^−4^	0.381
ko00500	Starch and sucrose metabolism	57	1	0.002	0.491
ko00250	Alanine, aspartate, and glutamate metabolism	29	2	0.002	0.263
ko01200	Carbon metabolism	110	3	0.002	0.192
ko00052	Galactose metabolism	30	1	0.004	0.538
ko00591	Linoleic acid metabolism	8	2	0.041	0.299
ko00910	Nitrogen metabolism	18	1	0.048	0.174
ko00430	Taurine and hypotaurine metabolism	3	2	0.285	0.023
ko00232	Caffeine metabolism	1	2	0.466	0.023
ko00230	Purine metabolism	30	8	0.639	5.833 × 10^−5^
ko00240	Pyrimidine metabolism	17	4	0.757	0.046
ko00660	C5-branched dibasic acid metabolism	1	3	0.968	0.006
ko00630	Glyoxylate and dicarboxylate metabolism	36	3	0.006	0.039

**Table 3:** Primer sequence of candidate genes.

Gene Short Name	Forward Primer Sequence	Reversed Primer Sequence
*LOC*103633247	GCCCATATCACTCCACCAAACCC	GACGGCGGCTTCTCCTCTCC
*LOC*100273222	ACACGACGCCGAGAGATCACC	CAACGCCGCTGTCTCCGATG
*gst2*	CAAGAAGCTGCTGTCGGAGA	GGTCCTCGAGCATGACCATC
*LOC*100193263	AAGTACATACGCTGCTCCAACTGC	AGCCTGCTCAACAATGTTCCTCAC
*LOC*103629384	GCAGCACCCAGGACGAAGTTAC	TGACAGCAACACCACCAGCAAG

## Data Availability

No new data were created or analyzed in this study.

## Supplementary Materials

The following supporting information can be downloaded at: https://www.mdpi.com/article/10.3390/ijms252212273/s1.
