# Supplementary material for: A Multi-Omics View of Maize’s (Zea mays L.) Response to Low Temperatures During the Seedling Stage
Source: Int J Mol Sci. 2024 Nov 15;25(22):12273. doi: 10.3390/ijms252212273 (PMC11595045; doi:10.3390/ijms252212273)
Supplement: Supplementary file 1 [file ijms-25-12273-s001.zip › ijms-3259266-supplementary.pdf]

International Journal of
Molecular Sciences

A

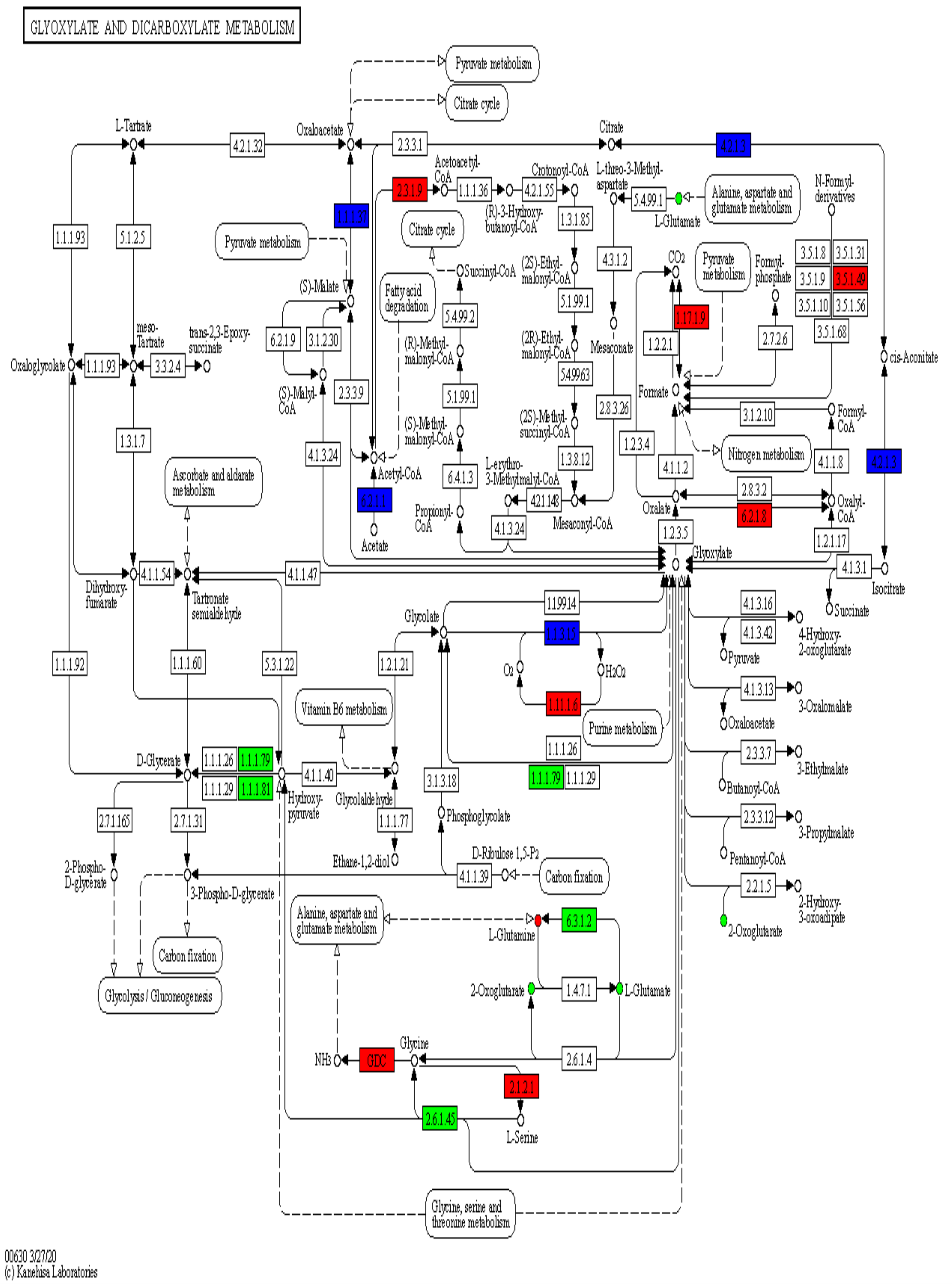

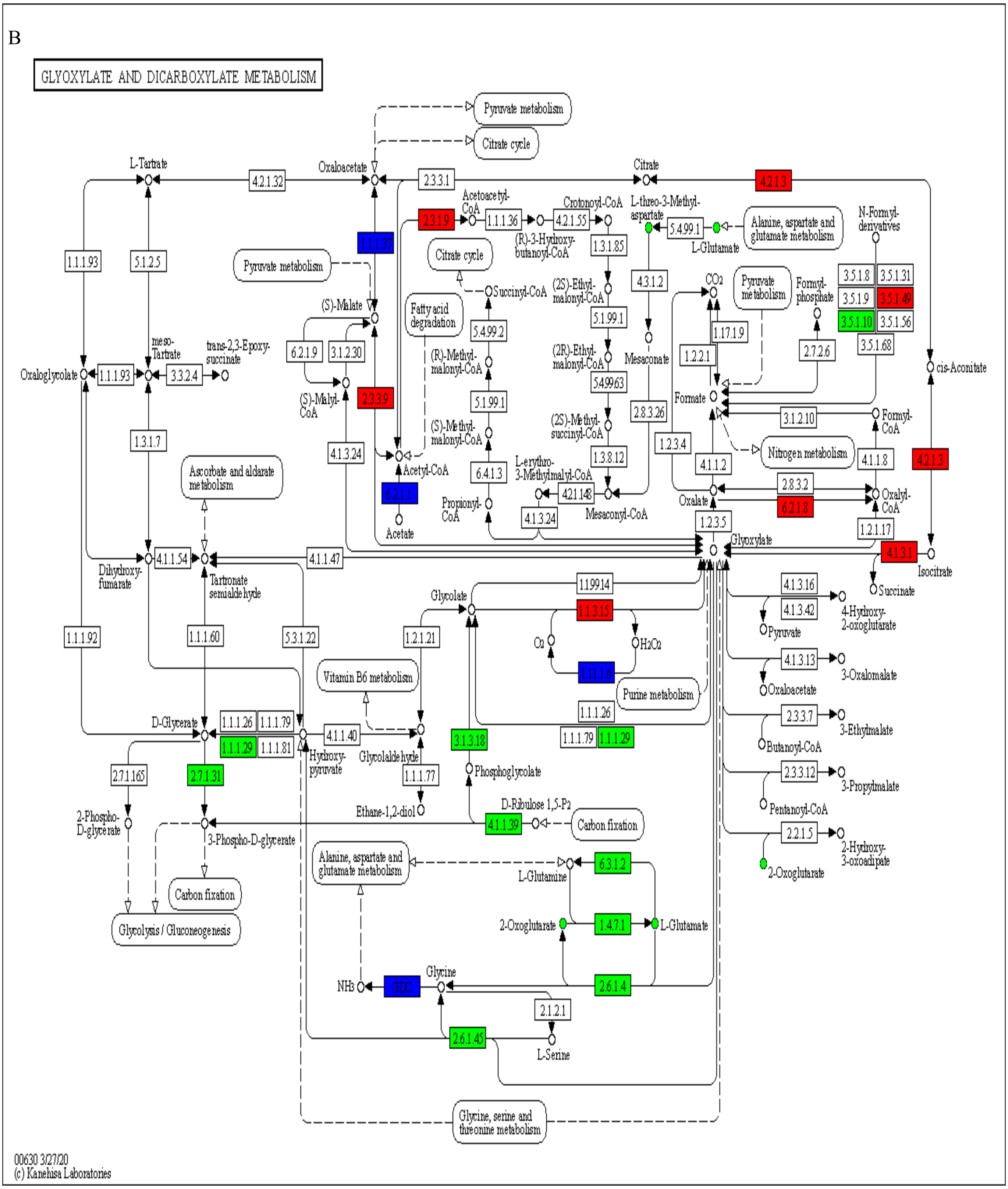

Supplementary Figure 1. Pathway diagram of Glyoxylate and dicarboxylate metabolism. Note: The red color in the figure indicates upregulation of genes/metabolites, the green color indicates downregulation of genes/metabolites, and the blue color indicates both upregulation and downregulation of genes and metabolites. Figure A shows the B144 Glyoxylate and dicarboxylate metabolism (ko00630) pathway diagram, and Figure B shows the Q319 Glyoxylate and dicarboxylate metabolism (ko00630) pathway diagram.
